# Multiparametric, Longitudinal Optical Coherence Tomography Imaging Reveals Acute Injury and Chronic Recovery in Experimental Ischemic Stroke

**DOI:** 10.1371/journal.pone.0071478

**Published:** 2013-08-07

**Authors:** Vivek J. Srinivasan, Emiri T. Mandeville, Anil Can, Francesco Blasi, Mihail Climov, Ali Daneshmand, Jeong Hyun Lee, Esther Yu, Harsha Radhakrishnan, Eng H. Lo, Sava Sakadžić, Katharina Eikermann-Haerter, Cenk Ayata

**Affiliations:** 1 Biomedical Engineering Department, University of California Davis, Davis, California, United States of America; 2 Neuroprotection Research Laboratory, Departments of Radiology and Neurology, Massachusetts General Hospital, Harvard Medical School, Charlestown, Massachusetts, United States of America; 3 Neurovascular Research Laboratory, Department of Radiology, Massachusetts General Hospital, Harvard Medical School, Charlestown, Massachusetts, United States of America; 4 Division of Drug Discovery Research, Korea Research Institute of Chemical technology, Yuseonggu, Daejeon, Korea; 5 Martinos Center for Biomedical Imaging, Department of Radiology, Massachusetts General Hospital, Harvard Medical School, Boston, Massachusetts, United States of America; 6 Stroke Service and Neuroscience Intensive Care Unit, Department of Neurology, Massachusetts General Hospital, Harvard Medical School, Boston, Massachusetts, United States of America; Tufts University, United States of America

## Abstract

Progress in experimental stroke and translational medicine could be accelerated by high-resolution *in vivo* imaging of disease progression in the mouse cortex. Here, we introduce optical microscopic methods that monitor brain injury progression using intrinsic optical scattering properties of cortical tissue. A multi-parametric Optical Coherence Tomography (OCT) platform for longitudinal imaging of ischemic stroke in mice, through thinned-skull, reinforced cranial window surgical preparations, is described. In the acute stages, the spatiotemporal interplay between hemodynamics and cell viability, a key determinant of pathogenesis, was imaged. In acute stroke, microscopic biomarkers for eventual infarction, including capillary non-perfusion, cerebral blood flow deficiency, altered cellular scattering, and impaired autoregulation of cerebral blood flow, were quantified and correlated with histology. Additionally, longitudinal microscopy revealed remodeling and flow recovery after one week of chronic stroke. Intrinsic scattering properties serve as reporters of acute cellular and vascular injury and recovery in experimental stroke. Multi-parametric OCT represents a robust *in vivo* imaging platform to comprehensively investigate these properties.

## Introduction

Experimental stroke research could benefit from new microscopic techniques to image disease during both its acute and chronic stages. In acute stroke, the penumbra is an area of brain tissue which is compromised but may possibly be salvaged. Unless perfusion is improved through thrombolysis, or cells are made more resistant to injury within hours, the penumbra dies as the infarct core expands over time. Macroscopically, diffusion and perfusion magnetic resonance imaging (MRI) [1] can assess tissue viability and flow concurrently, and thus help identify salvageable tissue at risk of infarction. In chronic stroke, over days to weeks, neurovascular responses underlie a transition from acute injury to delayed repair as the brain initiates plasticity and remodeling. An emerging and promising therapeutic strategy is to promote or augment these endogenous brain recovery mechanisms. Cell-based or pharmacologic therapies based on this strategy may have therapeutic time windows of days or longer [2], in contrast to thrombolytic therapy. Here again, longitudinal MRI provides insight into the spatiotemporal interplay of endogenous responses such as angiogenesis and axonal remodeling [3] that cannot readily be obtained from sectioning and histology at a single time point. However, microscopic imaging methods analogous to MRI are lacking.

A number of optical techniques have been applied in experimental stroke research [4]. Laser speckle flowmetry was a key innovation [5] that enabled the visualization of relative changes in cerebral blood flow (CBF), which confirmed the step-wise expansion of areas with a severe flow deficit in response to successive peri-infarct depolarizatons [6]. However, relationships of depolarization, repolarization or lack thereof, cellular damage, and ultimately cell death could not be directly assessed with laser speckle flowmetry. Furthermore, laser speckle flowmetry measures relative CBF [7], not absolute CBF, although the latter is more closely related to cell viability thresholds [8]. Two-photon microscopy [9] was the first technology to perform cellular imaging at depths of several hundred µm in the living brain, and has significantly impacted neuroscience research over the past 15 years. Recently, two-photon microscopy has emerged as an *in vivo* method to investigate changes in hemodynamics [10]–[12] and synaptic networks [13]–[16] in ischemic stroke and spreading depression [17], [18]. However, while two-photon microscopy achieves subcellular spatial resolution, the imaging speed, penetration depth, and field-of-view are limited. Although red blood cell (RBC) flux can be measured in individual capillaries [19], acquiring flow information over a large field-of-view required for assessing typical stroke models is cumbersome. While autoradiographic techniques [20] can provide high resolution spatial information on flow and actively metabolizing tissue, they only offer a single snapshot in time, since they require animal sacrifice and histology. Positron emission tomography (PET) can quantify hemodynamics and oxygenation dynamically [21], but its resolution is limited. Therefore, an unmet need in experimental stroke is the capability to comprehensively image the penumbra in small animal models with high spatiotemporal resolution.

Optical Coherence Tomography (OCT) possesses the capability to image over fields-of-view of several millimeters with micron-scale resolution, and thus may fulfill this need. Previously, optical micro-angiography was used for longitudinal imaging of revascularization after brain trauma [22]. A phase variance technique, termed “Doppler OFDI,” was used in tumor models to investigate vascular morphology, using cell scattering as a complementary indicator of viability [23]. The goal of the present study was to identify correlates of injury and recovery by multi-parametric OCT cortical imaging of two common stroke models; transient filament middle cerebral artery occlusion (fMCAO) and permanent distal middle cerebral artery occlusion (dMCAO). Using a glass coverslip-reinforced, thinned-skull cranial window preparation [24] that enabled longitudinal optical microscopy without excessively perturbing cortical physiology, we examined CBF, capillary perfusion, vessel diameter, tissue infarction, and capillary density longitudinally during experimental stroke and recovery in the mouse brain. The optical techniques presented here are analogous to multi-parametric MRI [25], [26], with the advantage of microscopic resolution. In the future, this imaging platform could be used to assess the impact of genetic mutations and therapies in cerebrovascular disease models, and could have applications in other fields such as tumor biology.

## Methods

### Experimental Procedures

All animal procedures were reviewed and approved by the Subcommittee on Research Animal Care at Massachusetts General Hospital, where these experiments were performed. Two commonly used experimental stroke models, transient fMCAO (filament middle cerebral artery occlusion, N = 4) and permanent dMCAO (distal middle cerebral artery occlusion, N = 4), were investigated with longitudinal optical imaging. The fMCAO model was used to study hemodynamic changes and their relationship to tissue scattering acutely, whereas the dMCAO model was used to study remodeling after one week. Additionally 3 mice with no experimental stroke were imaged. In total, for this study we imaged 15 hemispheres of 11 animals. Local anesthetic lidocaine 2% was injected before the beginning of all surgical procedures. For non-terminal surgeries, buprenorphine was administered. Additionally, during imaging, global anesthesia was maintained with isoflurane (between 1–1.5 v/v %), and mice were maintained at 37°C with a homoeothermic heating blanket controlled by a rectal thermometer. Cannulation of the femoral artery for blood gas, heart rate, and blood pressure monitoring was only performed before non-survival imaging experiments to minimize morbidity. Otherwise, anesthetic depth was titrated to maintain a breathing rate of about 1 Hz, which, based on our experience leads to physiological blood gas levels (Pa_O2_ = 80–120 mm Hg, pH = 7.35–7.45, Pa_CO2_ = 35–45 mmHg). However, isoflurane is a vasodilator and inconsistent depths of anesthesia may introduce variability in vasoreactivity between imaging sessions. Variable heart rate and blood pressure represent additional physiological factors that may alter CBF during survival experiments.

#### Thinned-skull cranial window preparation

Thinned-skull, glass coverslip-reinforced cranial windows were created in male, 2–4 month old C57BL/6 mice (N = 11) from Charles River Lab, enabling imaging of the boundary between middle cerebral artery (MCA) and anterior cerebral artery (ACA) supplied territories. Twenty-four hours before obtaining baseline images, cranial windows were created and animals were allowed to recover overnight. Following baseline imaging, MCAO was performed. For three of the four fMCAO mice and one of the four dMCAO mice, symmetric, bilateral cranial windows were created; in all other mice a single cranial window was created. Hemispheres contralateral to the occlusion served as additional controls. While homotopic reorganization has been suggested after stroke in rats [27], recent work has demonstrated that the contralesional cortex remodeling may not play a significant role in post-ischemic plasticity in mice [28].

To perform the cranial window surgery, the scalp and periosteum were reflected laterally, and the area of skull at the center of the parietal bone was thinned using a dental burr until soft and transparent (∼15–50 µm thickness). Cyanoacrylate cement (ND Industries, MI USA) was used to attach a coverslip to the skull for stabilization. The coverslip (Fisher Scientific) was 5 mm in diameter, and the usable imaging area was consequently approximately 3 mm x 3 mm, with the skull being thicker at the edges of this region. The infarct was visible through the imaging window for the fMCAO model, but was situated at the lateral edge of the imaging window for the dMCAO model.

Occasionally the skull thinning procedure caused bruising of the dura. Dilation of dural vessels at approximately 1 week, was also present in contralateral hemispheres of the experimental stroke group and in control animals without stroke. Therefore, dural vessel dilation was likely not related to the experimentally-induced ischemia. Additionally, skull regrowth slightly degraded the resolution over weeks [24]. However, we did not observe any differences in cerebral blood flow in control animals (i.e., skull thinning with no stroke) at 1 week compared to baseline, suggesting that the loss in resolution did not confound CBF quantification. Moreover, the skull regrowth did not impair our ability to visualize capillaries, even after weeks.

#### Transient fMCAO model

In three mice, fMCAO was performed on the right hemisphere and, in one on the left hemisphere. First, baseline imaging of both hemispheres was performed. Then, mice were placed in a prone position and the common carotid artery was exposed by a midline neck incision of 1 cm. A ligature was placed around the external and common carotid arteries. A loose suture was placed distal to the common carotid occlusion. An incision was made between the two sutures through which a silicone coated filament was advanced to the origin of the middle cerebral artery. After imaging only the ipsilateral hemisphere during 60 minute fMCAO, the filament was withdrawn. The surgical wound was sutured with monofilament suture material. Imaging of both hemispheres was performed again approximately 30 minutes and 60 minutes after filament withdrawal.

During the acute stroke imaging session, the cortical surface was monitored by simultaneous CCD imaging under green light illumination (λ = 570 nm +/−5 nm) for possible signs of spreading depolarizations. Surrounding the ischemic core, peri-infarct spreading depolarizations cause dramatic vasoconstriction, resulting in a decrease in total hemoglobin ([HbT]) and an increase in reflectance at the isosbestic wavelength λ = 570 nm [29]. When imaged with multi-parametric OCT, peri-infarct spreading depolarizations result in lower dynamic scattering due to reduced RBC content and reduced blood flow, along with increased static scattering from dendritic beading, organelle swelling, and intracellular water accumulation [30]. While peri-infarct spreading depolarizations almost certainly did occur throughout the experiment, simultaneous reflectance imaging confirmed that none occured during the OCT data acquisition, performed within a time span of a few minutes. Thus we confirmed that the OCT data was not confounded by spreading depolarizations concurrent with data acquisition.

#### Permanent dMCAO model

After baseline imaging, a burr hole (3 mm diameter) was drilled under saline cooling in the temporal bone overlying the distal MCA. The dura was kept intact. The exposed artery was occluded with microbipolar coagulation where it crosses the inferior cerebral vein. Repeat imaging was performed one week after permanent distal middle cerebral artery occlusion (dMCAO). The infarcts for the dMCAO model were typically situated lateral to the imaging window.

### System Description

**Figure 1:**
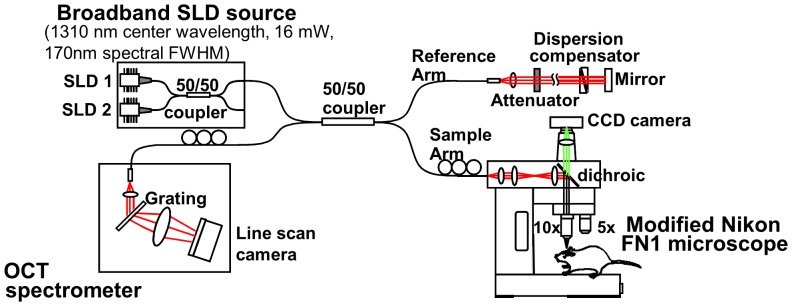
Schematic of spectral/Fourier domain OCT system and microscope. A dual-superluminescent diode (SLD) light source achieved an axial resolution of 3.5 µm in tissue. The OCT scanning system was built on a modified Nikon FN1 microscope platform, with a CCD camera and dichroic mirror for simultaneous viewing of the brain under visible light (570 nm ±5 nm).

As shown in Figure 1, a 1310 nm spectral/Fourier domain OCT microscope was constructed for *in vivo* imaging of the mouse cerebral cortex. The light source consisted of two superluminescent diodes combined using a 50/50 fiber coupler to yield a bandwidth of 150 nm. The axial (depth) resolution was 4.7 µm in air (3.5 µm in tissue). The power on the sample was 4 mW, and the sensitivity was 103 dB. A spectrometer with a 1024 pixel InGaAs line scan camera operated at 47,000 axial scans per second. Either a 5× objective or a 10× objective was used, yielding a transverse resolution of either 7.2 or 3.6 µm, respectively.

Mice were fixed in a custom mounted stereotaxic frame that enabled adjusting head tilt along the mediolateral axis. The acquisition times for the OCT angiogram and Doppler protocols were approximately 2 minutes each. The imaging field-of-view was situated above the MCA-ACA watershed region. Angiography was performed with both 7.2 µm and 3.6 µm transverse resolution, while Doppler OCT was performed with only 7.2 µm transverse resolution. The scan protocols and data processing are discussed in more detail below. Including time required for alignment (head tilt, translation, and focal depth), OCT imaging sessions required approximately 30 minutes per hemisphere.

### Data Acquisition and Processing

#### Angiography

**Figure 2:**
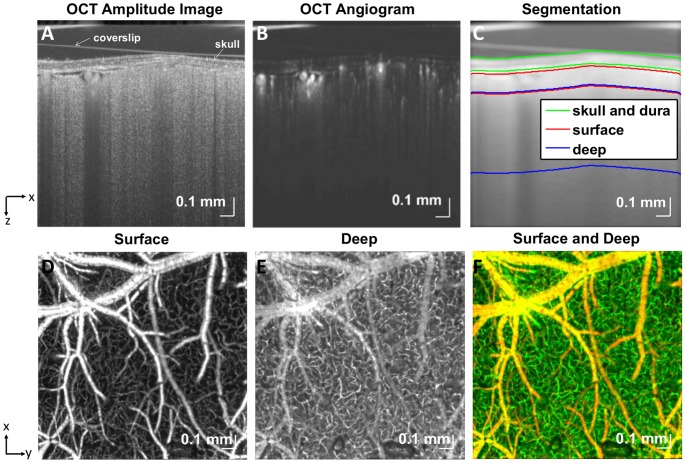
OCT angiography enables depth-resolved imaging of capillary perfusion. (A) Raw OCT image (logarithmic scale), acquired with the 10× objective, with the skull and coverslip labeled. (B) Normalized OCT angiogram (linear scale). (C) Segmentation of the image into three regions comprising the skull and dura, surface vessels (<100 µm depth), and deep vessels (>100 µm depth). (D) Surface vasculature map shows pial vasculature as well as diving arterioles and ascending venules. (E) Deep vasculature map shows perfusion in capillary beds. “Tails” from superficial vessels also appear in the map of deep vasculature, due to multiple scattering and motion correction errors. (F) Combination of surface and deep vasculature maps (surface - red channel, deep - green channel).

Similar to power Doppler ultrasound [31], OCT angiography estimates the scattering from moving red blood cells (RBCs). The OCT angiography scanning protocol acquired frames with 512 axial scans repeated twice at each transverse y location [32], at a total of 5120 transverse y locations. OCT angiography was performed over a cortical surface area of 3 mm x 3 mm (7.2 µm transverse resolution) or 1.5×1.5 mm (3.6 µm transverse resolution). In order to process data, high-pass filtering was performed along the slow axis of repeated B-scans at the same transverse location. Angiography requires detection and not quantification of motion; hence, angiograms were not adversely affected by aliasing due to the low sampling rate. The procedure for angiogram generation is shown in Figure 2. As previously described, the OCT complex signal can be divided into static and dynamic scattering components [33]. The power in the dynamic scattering component of the complex OCT signal is proportional to the scattering from red blood cells. Therefore relative changes in dynamic scattering are analogous to total hemoglobin measurements typically obtained from optical intrinsic signal imaging [34], often referred to as [HbT] in the literature. Some caveats to be noted are residual sensitivity to velocity changes if the power estimation procedure is imperfect, orientation dependence of scattering from RBCs [35], and the nonlinear relationship between hematocrit and scattering [36]. However, with these caveats in mind, dynamic scattering can be assumed to be monotonically related to RBC content. The OCT angiograms are useful in investigating compartment-resolved vessel tone and functional “recruitment” or “rarefaction” of perfused capillaries. One disadvantage of the OCT angiogram method is its motion sensitivity. Thus, even regions of non-vascular tissue will be detected if there is excessive motion. For instance, we frequently observed peri-arterial tissue regions as brighter in the angiogram due to pulsatility-induced motion artifacts. When arterial pressure was reduced during hypoperfusion, these artifacts disappeared.

#### Diameter

Using a MATLAB graphical user interface, vessel diameters were manually calculated in arteries, veins, and collaterals from the angiograms. By careful alignment of the animal it was possible to quantify diameter of the same vessel location at different time points for up to one week. The identities of vessels (artery, vein, or collateral) were determined from the OCT angiogram based on connectivity with larger vessels of a known type as well as morphology. Arterial collaterals were characterized by a more tortuous morphology than other arteries. We further confirmed vessel identities with Doppler OCT flow direction.

#### Flow

**Figure 3:**
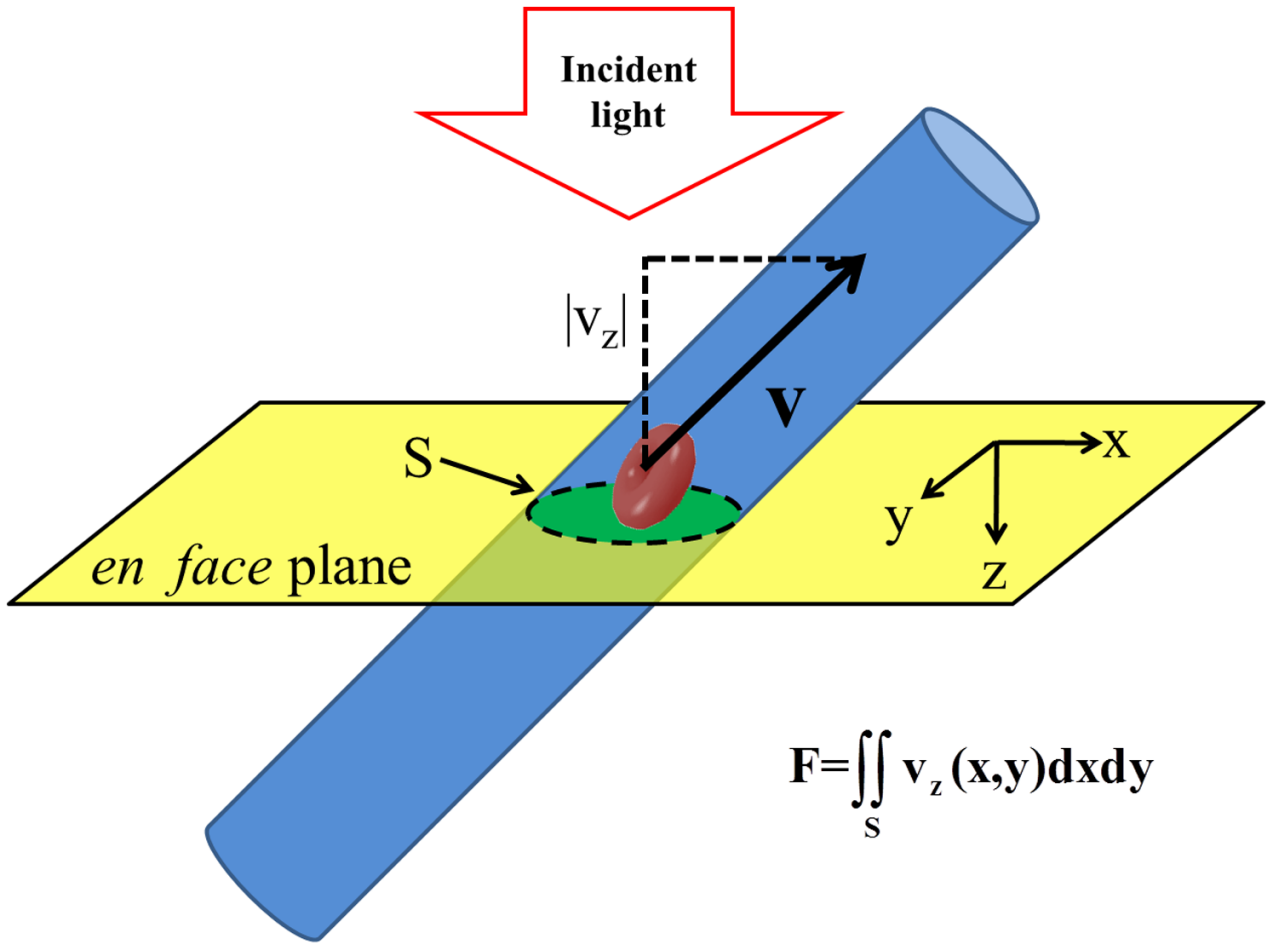
*En face* integration method quantifies flow without requiring explicit knowledge of vessel angle. An ascending vein with flow towards the incident probe beam is bisected by the en face plane. A red blood cell is shown, with a velocity vector given by **v**, while the magnitude of the velocity axial (z) projection is given by |v_z_|. Flow is obtained by integrating the axial projection of velocity over the *en face* plane.

As shown in Figure 3, flow was calculated from Doppler OCT data by integration of the velocity axial (z) projection in the en face (xy) plane [37]. This procedure is based on the assumption that velocity represents an incompressible (zero divergence) vector field. The en face integration procedure can be applied to pial arteries and veins provided that the vessel is angled and axial velocity projection is sufficient for detection. Thus, flow in individual vessels can be calculated and followed over time in an angle-independent fashion. However, without assumptions about the regions supplied or drained by these vessels, no information about nutritive supply (i.e., flow in mL/100 g/min) is obtained. Pial collaterals, which are numerous in the rat and mouse [38], confound the ascription of flow in specific pial vessels to particular parenchymal regions.

By contrast, flow in *diving* arterioles and venules can be assigned to well-defined cortical regions [10], [39]. Hence, in order to determine nutritive blood supply, flow is integrated over diving arterioles and ascending venules draining a given cortical surface area, yielding units of µL/mm^2^/min. To determine CBF in absolute units, this quantity is normalized to the estimated cortical mass corresponding to this surface area. Estimation of the cortical mass requires assumptions about cortical thickness (average over field-of-view of ∼1.5 mm [40]) and density (1.05 g/mL [41]). To enable comparisons of our data with a wide range of literature, flow values are presented using dual axes with units of µL/mm^2^/min and mL/100 g/min.

**Figure 4:**
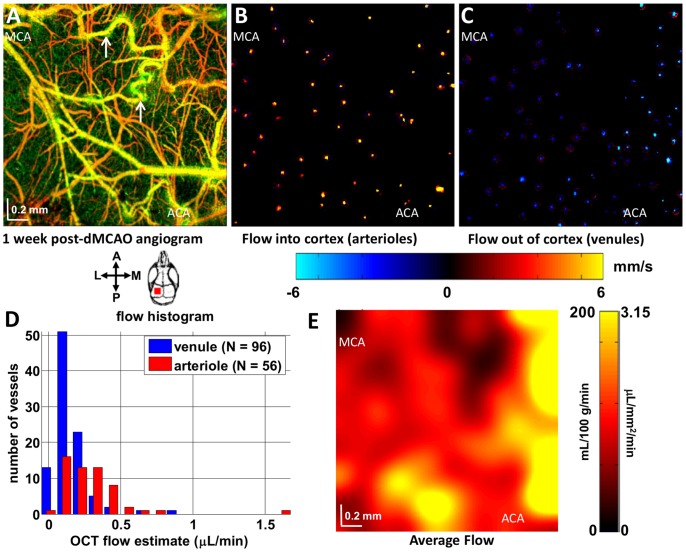
High resolution cortical flow mapping is achieved by calculating flow values in individual ascending venules and diving arterioles. (A) OCT angiography, after one week permanent dMCAO, in the MCA-ACA boundary region is shown, with prominent collateral growth (white arrows). Two-dimensional maps showing the axial projection of velocity in vessels either supplying (B) or draining (C) the cortex were created. (D) A flow histogram showed that venular locations (flow draining the cortex) were more numerous than arteriolar locations (flow supplying the cortex), but that the magnitude of flow per venular location was smaller. Thus, absolute cortical blood flow calculated from summing over all venules was 110 mL/100 g/min, while the cortical flow calculated from summing over all arterioles was 126 mL/100 g/min. (E) A spatial map of flow was formed by averaging arteriolar and (inverted) venular flow maps, and blurring with a 2-D Gaussian function with a half-width at half maximum of 0.2 mm.

To illustrate the procedure for flow calculation, Figure 4A shows an OCT angiogram, obtained from a thinned-skull cranial window preparation in the mouse, one week after permanent distal MCA occlusion. The pial and dural vessels are colored yellow/orange, while the deep vessels are colored green. 3D Doppler OCT data sets were acquired over a cortical surface area of 2.5 mm x 2.5 mm with a resolution of 7.2 µm. The Doppler OCT protocol consisted of 4 consecutive volumes of 256 frames with 4096 axial scans each [42]. Each volume required approximately 26 seconds, hence the Doppler OCT scanning protocol required about 2 minutes to complete. Doppler OCT velocities were computed using adjacent axial scans along the fast axis of the frame; hence the maximum measurable velocity axial projection, determined by the axial scan rate, was 11.4 mm/s. Care was taken to place the focus approximately 100 µm below the cortical surface, using the CCD camera view as a guide for the focal plane location. Using another custom-designed MATLAB graphical user interface for flow calculations, flow was rapidly calculated in individual vessels. For each diving or ascending vessel, a location was chosen near the cortical surface, typically within the first 150 µm, where the vessel angle was sufficient to yield a measurable Doppler shift. Measuring vessels too close to the surface resulted in insufficient Doppler shifts, while measuring vessels too deep resulted in low signal-to-noise ratios. Although for each vessel the selected depth was different, it was possible to construct an *en face* flow map from all selected vessels showing the flow into the cortex (Figure 4B) and the flow out of the cortex (Figure 4C). The histogram of individual vessel flows is shown in Figure 4D. A total of 96 venules drained the cortex, while only 56 arterioles supplied the cortex. Due to the higher average flow per arteriole, the total arteriolar flow was 126 mL/100 g/min, while the total venular flow was 110 mL/100 g/min. Assuming that the cortex is supplied and drained at the cortical surface, the total flow entering and leaving the cortex via the surface should be theoretically equal in steady state. Absolute flow measurements and maps were based on the average of arteriolar and (inverted) venular flow maps. It has previously been shown that Doppler OCT only measures velocity axial projections above a certain threshold [43]. Therefore, in practice, due to the larger arteriolar velocities, measured arteriolar flow typically exceeded measured venular flow. However, venules are more numerous than arterioles and thus may be more spatially specific. In order to blur out discrete sources and sinks in the flow maps, we convolved them with a 2-D Gaussian function with a half-width at half maximum of 0.2 mm, including reflections at boundaries (Figure 4E). The resulting composite map roughly depicts the spatial distribution of flow, without detailed information about supply and drainage territories.

#### OCT signal slope analysis

Because energy is required to maintain cellular integrity and structure, energy deficits are accompanied by changes in cellular morphology and light scattering. We noted that the OCT signal penetration was reduced after reperfusion in the region destined for infarction, consistent with well-known increase in scattering after anoxic depolarization in ischemic tissue [44]. To interpret the changes in OCT signal characteristics we observed, we employed a simplified single scattering model to describe the OCT signal amplitude as a function of sample depth z, as shown below [45].
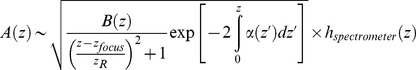
(1)where B is the backscattering, z_focus_ is the depth of the focus, z_R_ is the Rayleigh range, α is the depth-varying attenuation coefficient, and h_spectrometer_ is the roll-off function of the spectrometer. By taking the natural logarithm of the above expression, we obtain:



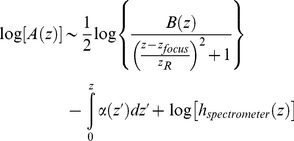
(2)We placed z_focus_ at the same depth during each imaging session, as described above. We also ensured that the brain surface was located at the same path length delay during each imaging session. Thus, we attribute changes in OCT signal slope characteristics to the middle term in the above expression, involving the depth-varying attenuation coefficient α.

**Figure 5:**
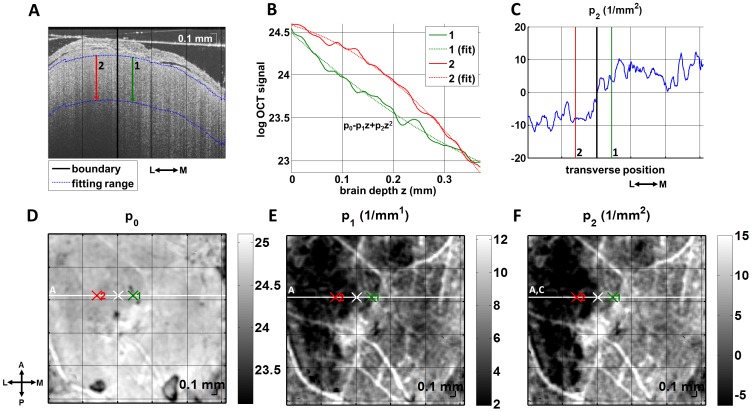
Analysis of acute cellular scattering changes after transient fMCAO. **(**A) Cross-sectional image (60 minutes after reperfusion) showing reduced OCT signal penetration on the lateral side compared to the medial side. Two exemplary axial lines (red and green) are chosen for analysis of the OCT signal. (B) Plots of logarithmic OCT signal vs. depth along these lines, after averaging (80 µm transverse, 35 µm axial), along with second-order polynomial fits at the two points labeled 1 (non-infarct) and 2 (infarct). (C) Plot of the second-order coefficient from the polynomial fit (p_2_), showing a transition in the curvature from concave down (lateral side) to concave up (medial side). (D–F) Images of zeroth, first, and second order polynomial coefficients from the polynomial fit. In the second-order coefficient image (F), a clear transition in scattering characteristics is seen.



After segmenting out the skull, we performed a second-order polynomial fit on the OCT axial signal profile between a depth range of 0 and 0.375 mm, as shown in Figure 5,

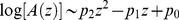
(3)As shown in Figure 5, the shape of the OCT logarithmic signal profile is concave down in the infarct, and therefore the sign of the second-order coefficient p_2_ clearly delineates the infarcted tissue. Other metrics consistent with our observations, such as OCT signal penetration depth, or the OCT signal from a fixed depth beneath the cortical surface, also demarcated infarcted tissue.

#### Histology

At the end of OCT imaging period, approximately 2 hours after reperfusion, mice with transient fMCAO were decapitated and brains were harvested and snap-frozen in −40°C 2-methylbutane. Twenty micron thick cryosections (100 micron intervals) were collected on histological slides and immunostained using an anti-MAP2 primary antibody (Chemicon, 1∶400 in 3% normal horse serum, overnight at 4C). Sections were then incubated with a horse anti-mouse secondary antibody-FITC conjugated (Jackson ImmunoResearch, 1∶200 in PBS) for one hour at room temperature, and then counterstained with Hoechst (Sigma, 1∶1000, in PBS) and coverslipped. To study cellular morphology, adjacent sections were stained with Cresyl Violet Acetate (0.1%). Images were acquired using a Nikon TE-2000 microscope equipped with epifluorescence illumination.

## Results

Below, we describe and quantify hemodynamic and cellular changes in the models of acute and chronic stroke using multiparametric OCT.

### Transient fMCAO Model

A transient fMCAO model was used to study the relationship between hemodynamics and tissue scattering changes in acute stroke.

#### Capillary non-perfusion

Capillary perfusion was investigated with OCT angiography at baseline, during fMCAO, and after filament withdrawal. In all mice, during fMCAO there was a clear border between capillary-perfused tissue and capillary non-perfused tissue, where “perfusion” is defined by the presence of moving blood cells. After filament withdrawal, the very same capillaries which were absent in the angiograms during occlusion typically reappeared.

**Figure 6:**
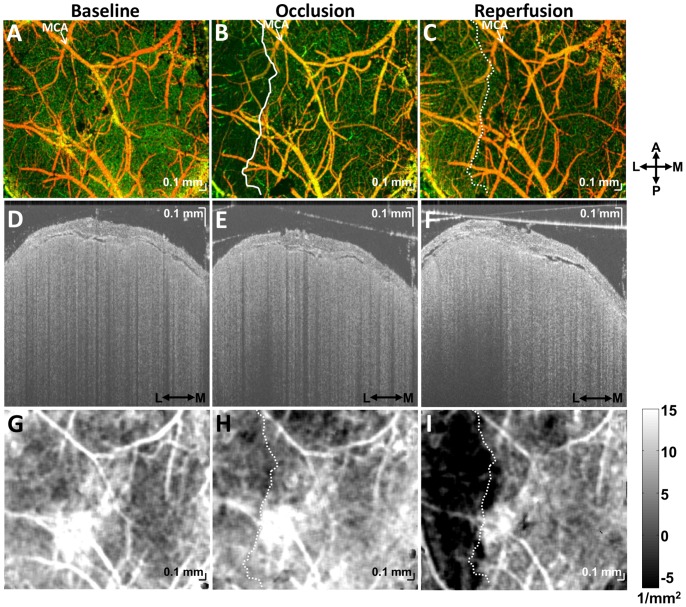
Capillary non-perfusion during fMCAO predicts cellular scattering changes leading to eventual infarction. (A–C) OCT angiograms at baseline, during fMCAO, and 60 minutes after filament withdrawal. During fMCAO (B), a capillary non-perfused region is apparent, as demarcated with a solid white line. (D–F) OCT cross-sectional images on a logarithmic scale, with minor differences in alignment, show changing signal characteristics in the lateral portion of the cranial window after reperfusion (F). (G–I) Images of the curvature of the OCT signal vs. depth at baseline, during fMCAO, and 60 minutes after reperfusion show this evolution. In (H–I), the capillary non-perfusion boundary from (B) is shown as a dotted white line. In particular, the tissue with anomalous scattering properties (i.e., the log OCT signal vs. depth is concave down) after reperfusion corresponds well to the non-perfused tissue during fMCAO. No comparable changes were observed in the contralateral hemisphere.

Figure 6A–C shows angiograms at baseline, during transient fMCAO, and after filament withdrawal. The entire cranial window contained capillaries perfused with moving blood cells at baseline, as shown in Figure 6A. However, during MCA occlusion, a clearly defined region devoid of perfused capillaries was observed in the lateral portion of the cranial window, as outlined with a solid white line in Figure 6B. Sixty minutes after filament withdrawal, RBC perfusion returned to the capillaries in this region, as shown in Figure 6C.

#### Capillary non-perfusion and infarction

We simultaneously investigated changes in cellular scattering during these periods as shown in Figure 6D–F. In particular, we observed a reduction in OCT signal penetration depth 60 minutes after reperfusion (Figure 6F). Comparable changes were also observed 30 minutes after reperfusion (not shown). We found that plotting the second order coefficient of a polynomial fit of the natural logarithm of the OCT signal vs. depth demarcated the region with reduced OCT signal penetration (see Figure 5). The boundary between capillary perfused and capillary non-perfused areas is shown as a dotted white line in Figure 6H–I. Figure 6I shows clearly that the cortical scattering properties on the lateral side are changed (the OCT logarithmic signal vs. depth profile has negative curvature over the first 375 µm). Moreover, capillary non-perfusion (dotted white line) during occlusion correlates spatially with the eventual development of altered scattering properties.

**Figure 7:**
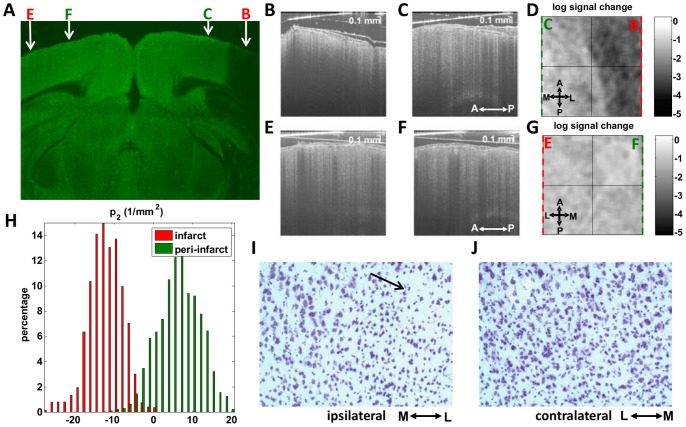
Correlation of acute cellular scattering changes after transient fMCAO with MAP2 immunohistochemistry, approximately two hours after reperfusion. (A) Coronal section shows a clear delineation of the lesion, with absent MAP2 immunoreactivity. Sagittal OCT cross-sections in the infarct (B) and peri-infarct (C) regions show differences in signal characteristics. (D) When the logarithmic signal change over the first 250 microns of cortical tissue is displayed *en face*, a clear border is observed, delineating the infarct. The OCT signal characteristics of the contralateral cortex (E–G) are comparable to those of the peri-infarct cortex. (H) Curvature differences, determined from Equation 3, are also observed between infarct and peri-infarct cortical regions, as suggested by Figure 5. (I–J) Aberrant cortical cellular morphology, visualized approximately 2 hours after reperfusion by Cresyl Violet near the ipsilateral lesion boundary (black arrow), may partially account for the observed scattering changes.

To further understand changes in scattering properties, we directly co-registered our OCT cross-sections with Hoechst, MAP2, and Cresyl Violet stained-histological slices. MAP2 is localized mainly to the dendrites of neurons, and absent or attenuated immunoreactivity of MAP2 is a sensitive marker of ischemic damage after transient focal ischemia [46]. We co-registered MAP2 stained sections with *in vivo* OCT images from the same brain based on the location of the thinned skull window in Paxinos and Watson coordinates [40]. OCT cross-sections could therefore be co-registered with corresponding MAP2 photographs (Figure 7A). In lesional and perilesional cortical regions, defined based on the MAP2 sections, scattering properties were found to differ (Figure 7B–D). Contralateral signal properties (Figure 7E–G) were similar to those in the perilesional cortex. As suggested by Figure 5, infarction was characterized by a change in the curvature of the OCT logarithmic signal profile (Figure 7H). Cresyl Violet depicted a transition to aberrant cellular morphology at the ipsilateral lesion boundary (Figure 7I–J), potentially explaining the observed OCT signal changes.

#### Regional blood flow

**Figure 8:**
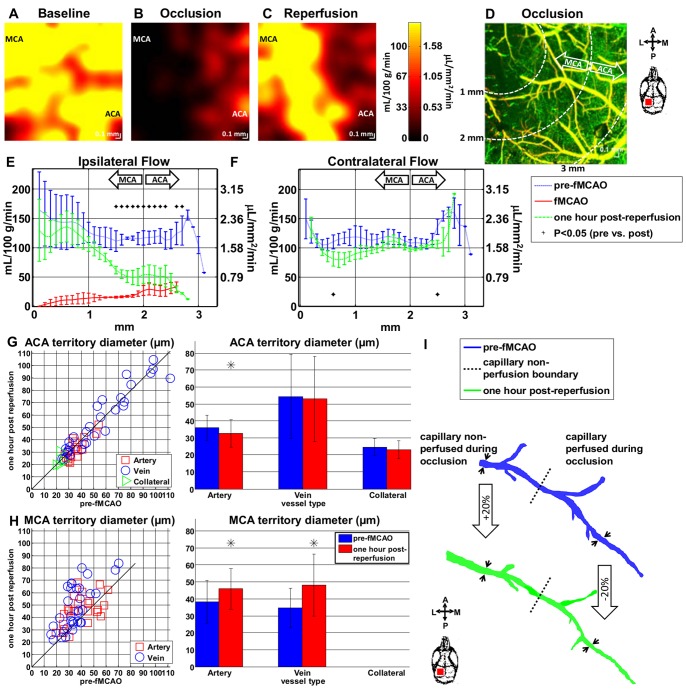
Spatially heterogeneous flow and diameter changes were observed during fMCAO and after reperfusion. Absolute flow maps show uniform flow at baseline (A), preferentially reduced MCA flow during occlusion (B), and restored MCA flow, with a persistent ACA flow deficit, after reperfusion (C). (D) An angiogram from the same animal during occlusion shows capillary non-perfusion in the lateral portion of the cranial window, presumably delineating the region destined for infarction, as suggested by Figure 6. (E–F) Regional blood flow was estimated as a function of distance between the MCA and ACA supplied territories. Flow was restored to baseline values on the MCA side after reperfusion, while a flow deficit persisted on the ACA side (E). These changes were not mirrored by the contralateral hemisphere (F). (G–H) Consistent with these results, vessels preferentially dilated in the MCA region, and constricted in the ACA region after reperfusion. (I) Remarkably, even along a single artery, both dilation and constriction were observed, depending on the earlier presence of nearby capillary non-perfusion.

Regional blood flow was measured in both MCA and ACA territories at baseline, during fMCAO, and after reperfusion in the ipsilateral hemisphere. Flow maps from a single representative animal are shown in Figure 8A–C, with the corresponding angiogram, acquired during fMCAO, in Figure 8D. Regional flow is plotted as a function of distance between the MCA and ACA territories for both the ipsilateral (Figure 8E) and contralateral (Figure 8F) hemispheres. As shown in Figure 8A,E there was no gradient in flow at baseline. During fMCAO flow dropped drastically, with the most dramatic reduction in the MCA region (Figure 8B,E). After reperfusion, flow recovered to a certain degree, with higher flow in the MCA region compared to the ACA region (Figure 8C,E). Blood flow in contralateral hemispheres showed no parallel changes after reperfusion (Figure 8F). Vessel diameters exhibited changes consistent with the observed flow changes, i.e. dilation in the MCA region (Figure 8G) and constriction in the ACA region (Figure 8H). The latter may have been the result of a prior peri-infarct spreading depolarization.

#### Impaired autoregulation

Vessel caliber measurements revealed evidence of autoregulation failure in the ischemic region destined to become infarcted. In particular, in contrast to the arteries in the ACA region, vessels in the MCA region showed marked vasodilation after reperfusion. This is consistent with the observation that post-reperfusion flow nearly recovered to baseline levels on the MCA side, but not the ACA side (Figure 8C,E). Even along a single vessel traversing the previously-defined perfusion/non-perfusion boundary, we observed spatially segregated constriction and dilation after filament withdrawal (Figure 8I). These contrasting observations may be explained by impaired control of vascular tone to regulate tissue perfusion in the ischemic MCA territory destined for infarction and/or persistent oligemia due to a prior spreading depolarization in the ACA territory.

### Permanent dMCAO Model

A permanent dMCAO model was used to study remodeling in chronic stroke.

#### Regional blood flow

**Figure 9:**
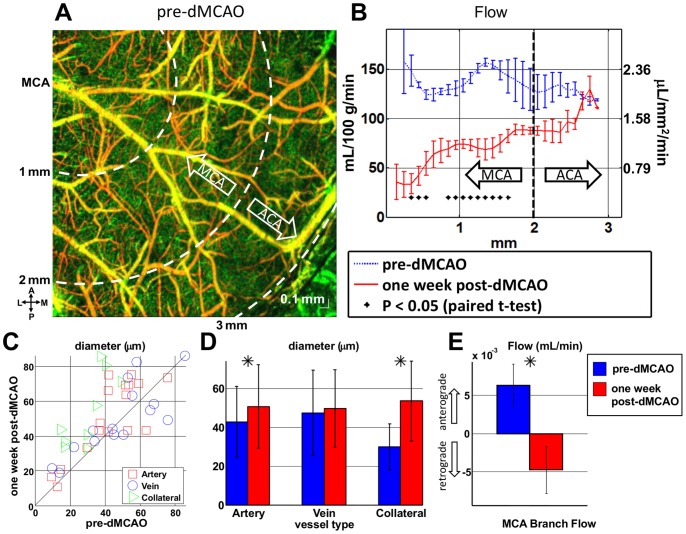
Remodeling in chronic stroke was investigated with a permanent dMCAO model. Blood flow was measured in both diving arterioles and ascending venules. (A) Regional blood flow was estimated as a function of distance between the MCA and ACA supplied territories. While flow was relatively uniform before occlusion, a gradient in flow was observed after one week permanent dMCAO. (B) Flow was reduced on the MCA side, while flow was preserved near the ACA side, showing the impact of the collateral ACA supply. (C–D) Diameter changes were evident in both MCA and ACA arteries as well as collaterals. (E) Doppler OCT revealed a reversal in flow direction in major MCA branches. This flow reversal is due to the large pressure drop in the occluded branches, and may be further enhanced by collateral growth and artery dilation.

At the one week time point, blood flow was measured in both diving arterioles and ascending venules (Figure 4). A gradient in flow from the middle cerebral artery (MCA) side to the anterior cerebral artery (ACA) side was visualized. Regional blood flow was estimated as a function of distance between the MCA and ACA supplied territories (Figure 9A). While flow was relatively uniform before occlusion, a gradient in flow was observed after one week dMCAO. Flow was reduced on the MCA side, while flow was preserved near the ACA side, showing the impact of the collateral ACA supply.

#### Arteriogenesis

OCT angiography revealed dramatic growth of surface collaterals after one week permanent dMCAO, including increases in diameter and tortuosity. While diameters of pial branches of the MCA, ACA, and collaterals increased significantly (Figure 9C–D) no statistically significant diameter changes in veins were detected. Previous studies have reported growth of surface collaterals stimulated by chronic elevation in shear stress [47].

#### MCA branch flow

Doppler OCT revealed a reversal in flow direction in major MCA branches (Figure 9E). This flow reversal is due to the large pressure drop in the occluded branches, and may be further enhanced by collateral growth and pial artery dilation.

#### Vascular remodeling

**Figure 10:**
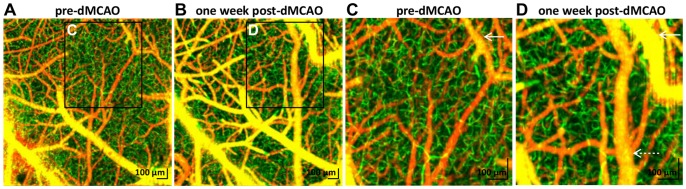
OCT angiography suggested remodeling in the border zone during distal MCAO. Angiograms were acquired over a 1.5 mm x 1.5 mm field-of-view with a transverse resolution of 3.6 µm before (A) and after (B) one week permanent dMCAO. (C–D) Zoomed images show pial collateral growth (solid white arrows), dural vessel dilation (dotted white arrow), and a more irregular capillary bed (green), suggesting angiogenesis.

High-resolution OCT angiography images revealed evidence of vascular remodeling in the border zone during chronic dMCAO (Figure 10). Significant collateral growth was observed (white arrows in Figure 10C–D). Extensive collateral growth was also observed at the same time point in Figure 4A (white arrows). Dilation of dural vessels, possibly related to trauma from the skull-thinning surgery, was noted (dotted white arrow in Figure 10D). Dural vessel dilation was also observed in contralateral hemispheres (not shown) and in control animals with thinned-skull cranial windows, but without experimental stroke. Notably, no obvious new pial vasculature was detected. The capillary bed had a more tortuous appearance after one week, suggesting remodeling and possible angiogenesis (Figure 10C–D). The angiography technique only detects perfused capillaries, and may miss new, immature capillary buds that are not yet perfused by blood cells.

### Conclusions

The cellular and hemodynamic status of cortical tissue *in vivo* can be determined from scattering signatures imaged by multi-parametric OCT. While two-photon microscopy achieves subcellular spatial resolution with a range of contrast mechanisms, the imaging speed, penetration depth, and field of view are limited. Conversely, while diffusion/perfusion weighted MRI or hemodynamic/metabolic PET can assess flow and tissue viability concurrently, much higher resolution imaging is desirable for mouse models. Multi-parametric OCT fulfills a niche between all of these modalities, depicting tissue architectural, but not subcellular, changes.

### Cellular Monitoring

Our data suggest that cellular scattering signatures can monitor the ischemic injury cascade leading up to infarction. In the normal rodent brain, we typically observe a higher OCT signal slope in layer I than layer II/III, causing the overall depth profile to be concave upwards. This may be related to higher cortical myelination in layer I [30]. However, in the capillary non-perfused tissue destined for infarction, this pattern eventually inverts (Figures 5–7), leading to a depth profile that is concave downwards. One possible explanation for this is that cell swelling and intracellular organelle swelling increase scattering, particularly in layer II/III. We also observed reduced OCT signal penetration in the infarcted region (Figure 7B–D), consistent with a scattering increase reported previously from necrotic tissue [23]. While our data support the assertion that scattering increases, the depth profiles further suggest that scattering changes may be heterogeneous and layer-specific, a possibility which can be further investigated using Optical Coherence Microscopy [30].

### Hemodynamic Monitoring

Our data illustrate the capability to longitudinally monitor CBF, capillary perfusion, vessel diameter, and capillary density. However, our Doppler algorithm may underestimate flow under conditions of severe hypoperfusion. Venules generally have smaller velocities, and hence, smaller velocity axial projections, than arterioles. This may lead to velocity thresholding and underestimation of venular flow under hypoperfused conditions; however venules are more numerous than arterioles and therefore offer more spatial specificity. Therefore, in this work, flow was estimated using the average of arteriolar and venular flow. This represents a simple method to quantify and map CBF with high spatial resolution (Figure 4E). Future work will determine whether cell viability thresholds based on Doppler OCT flow agree with those determined by other techniques such as PET.

In this study, vessel diameter was inferred from the width of the region with dynamic scattering blood contents [23]. Typically, vessel diameter is defined by the width of the plasma or distance between two edges of the vessel wall. Therefore, one possible confound is the Fåhræus–Lindqvist effect, which leads to the presence of a plasma cell-free layer near the wall that is depleted of RBCs. Due to this effect, the diameter inferred from blood cell scattering does not equal the true vessel diameter. As the size of the plasma cell-free layer is shear stress dependent, the apparent vessel diameter in the OCT angiogram could be influenced by changes in velocity and shear stress, even in the absence of dilation or constriction. Thus, measured diameter changes on the order of a micron or less should be interpreted with caution.

Capillary changes depicted by OCT angiography must likewise be interpreted with caution. The fact that capillaries can temporarily “disappear” (Figure 6B) and “reappear” (Figure 6C) suggests that OCT angiography is not a suitable technique for monitoring capillary-level angiogenesis under conditions of severe hypoperfusion. Capillaries may be collapsed and not perfused by blood cells, but otherwise intact. Such capillaries are effectively invisible to the OCT angiography technique. Moreover, immature endothelial buds that are not yet perfused with blood cells are also not detectable with OCT angiography. However, with these limitations in mind, Figure 10 shows a qualitatively more tortuous capillary bed one week after stroke, suggestive of angiogenic remodeling in the border region.

### Imaging Biomarkers that may Predict Infarction

Our imaging results suggest candidate biomarkers for eventual tissue infarction: capillary non-perfusion (Figure 6B), altered cellular scattering (Figure 6F, I), CBF deficit (Figure 8B, E), and impaired CBF autoregulatory capacity after reperfusion (Figure 8C, E, & I). During acute stroke, a capillary non-perfused region, corresponding spatially with eventual increases in cellular scattering (Figure 6), was observed. Interestingly, these scattering changes developed (Figure 6F, I) although capillary perfusion returned after filament withdrawal (Figure 6C). Moreover, the altered scattering properties correspond closely with the MAP2-defined lesion and aberrant cellular morphology (Figure 7), suggesting that the degree and duration of ischemia has exceeded the viability threshold. Possible physiological causes for the scattering changes include intracellular water accumulation, organelle swelling, and dendritic beading. There may also be a small contribution from increases in RBC content. Future investigations with shorter times to reperfusion will verify whether or not the duration of capillary non-perfusion indeed predicts eventual infarction.

Our data suggest preferentially increased blood flow and evidence of impaired vessel tone in the MCA territory (Figure 8H, I) after reperfusion. The recovery of blood flow to injured tissue with a presumably lower metabolic demand may be indicative of “luxury perfusion,” and has been observed in other experimental models [48], [49]. The reduced flow on the ACA side (Figure 8E) and corresponding vasoconstriction (Figure 8G, I) may be attributable to a prior peri-infarct spreading depolarization [6], [8]. These observations complement a previous study that showed preserved vessel tone in penumbral small arteries and penetrating arterioles after transient filament occlusion [50], and further suggest preferential loss of vasoreactivity closer to the stroke core.

Most compellingly, the candidate biomarkers discussed above might present at different locations in space and time. Spatial or temporal mismatches may delineate salvageable tissue which is at-risk but not yet destined for infarction, analogous to the well-known diffusion-perfusion mismatch [1]. This intriguing possibility will be the subject of future studies.

Moving from the acute to chronic stages, our results also shed light on potential mechanisms of flow recovery. During a focal stroke, flow in the pial arterial network passively reverses direction to supply the hypoperfused region [12]. In the chronic stages, arteriogenesis occurs, with collaterals doubling in size to actively supply even more flow to the hypoperfused territory (Figure 9C–D). Active collateral growth is likely caused by endothelial cell proliferation driven by flow reversals and persistent shear stress elevation caused by the *focal* pressure drop in the occluded MCA branch. Notably, in a mouse model of bilateral carotid stenosis leading to chronic *global* hypoperfusion, this dramatic collateral growth was markedly absent after one week (not shown). Beyond suggesting that both mechanisms are present, however, our results do not quantitatively address the degree of active versus passive flow redistribution. We also show anecdotal evidence of angiogenesis in chronic stroke (Figure 10), which is thought to supplement collateral growth in the border region [47].

In conclusion, longitudinal multi-parametric OCT provides absolute, quantitative hemodynamic indicators and markers of cellular status for comprehensive monitoring of ischemic injury and recovery. Scattering signatures of cellular and hemodynamic changes were identified during both injury and repair. This novel imaging platform has the potential to significantly accelerate translational pre-clinical studies.
